# Retrosplenial Cortex Codes for Permanent Landmarks

**DOI:** 10.1371/journal.pone.0043620

**Published:** 2012-08-17

**Authors:** Stephen D. Auger, Sinéad L. Mullally, Eleanor A. Maguire

**Affiliations:** Wellcome Trust Centre for Neuroimaging, Institute of Neurology, University College London, London, United Kingdom; National Institute of Mental Health, United States of America

## Abstract

Landmarks are critical components of our internal representation of the environment, yet their specific properties are rarely studied, and little is known about how they are processed in the brain. Here we characterised a large set of landmarks along a range of features that included size, visual salience, navigational utility, and permanence. When human participants viewed images of these single landmarks during functional magnetic resonance imaging (fMRI), parahippocampal cortex (PHC) and retrosplenial cortex (RSC) were both engaged by landmark features, but in different ways. PHC responded to a range of landmark attributes, while RSC was engaged by only the most permanent landmarks. Furthermore, when participants were divided into good and poor navigators, the latter were significantly less reliable at identifying the most permanent landmarks, and had reduced responses in RSC and anterodorsal thalamus when viewing such landmarks. The RSC has been widely implicated in navigation but its precise role remains uncertain. Our findings suggest that a primary function of the RSC may be to process the most stable features in an environment, and this could be a prerequisite for successful navigation.

## Introduction

The ability to navigate is critical for survival. As such, there have been decades of research exploring how environments are represented internally, the key components of these representations, and the brain regions that support them. From the outset of systematic studies of navigation, prominent features in an environment, known as landmarks, have been posited to play a role [1]–[3]. In some theoretical formulations, landmarks are cast as the very building blocks of environmental representations [2], [4], [5]. In others, such as the cognitive map theory, spatial relations between landmarks are regarded as the basis for a critical form of flexible navigation [6], [7], while even accounts that emphasise navigation via path integration (i.e. estimating current location based on the movements made since the last known location), acknowledge the role of landmarks in maintaining accuracy [8], [9].

Given their importance for navigation [10], what is it about landmarks that makes them so useful? This seems like an obvious question, however, the majority of experiments involving landmarks have focused on their use or presence during active navigation or other spatial tasks. By contrast, the properties of the landmarks themselves have received much less attention, yet understanding this may provide important clues about how environmental representations are formed and how navigation is supported. There is a relative dearth of information about landmark features because it has proved difficult to develop an agreed method for assessing landmark properties [11]. Characterisation of landmarks is a somewhat subjective process, and individual differences may contribute to the difficulty in deriving standardised landmark classifications. Several properties of landmarks have been highlighted as potentially important [12], including the permanence or stability of the landmark (i.e. the likelihood of the landmark being present), its usefulness for navigation (e.g. proximity to a decision point), and its visual features (e.g. size, salience, visibility).

With such difficulty establishing the key properties of landmarks, it is not surprising that the neural correlates of landmarks are not easily determined either. While there is a wealth of evidence from neurophysiological and lesion studies in animals, and neuroimaging and neuropsychological studies in humans for the brain areas involved in supporting navigation [13]–[17], scene processing [18]–[20], and representations of topographical features [21]–[23], findings have rarely been linked to specific landmark properties. There are a few exceptions; as noted above, the position of landmarks within an environment has been emphasised [12], [24], [25]. In animal studies, whether landmarks are positioned proximally or distally is thought to influence navigation and the control of place fields, with distal landmarks being particularly significant, perhaps because they do not appear to change too much when the animal moves ([6]; see [9], [10] for recent reviews). Currently there is not agreement about the neural substrates of proximal and distal landmark control [9]. In human fMRI studies, posterior parahippocampal cortex (PHC) has been shown to be particularly responsive to items (in this case toys) encountered at navigationally relevant decision points in a virtual reality museum [26]–[28]. Similar PHC activation has also been found for landmarks on real-world routes [29], although this latter study utilised permanent landmarks (buildings) at decision points and observed additional activity in retrosplenial cortex (RSC) and along the parietal-occipital sulcus.

As previously noted, an item’s size and permanence within the environment may also be important properties [12]. Interestingly, the combination of these two features was found to evoke a strong sense of space surrounding single acontextual objects (rendering them ‘space-defining’ - SD) even when imagined or viewed in isolation [30]. Outdoor SD landmarks as well as indoor SD objects were associated with increased activity in PHC. Moreover, further interrogation of these data revealed a selective response in RSC that was specifically linked to item permanence over and above that which was captured by the SD response alone (see unpublished data from [30] in Figure S1). These observations, combined with the greater sense of stability offered by distal landmarks [6], and the utility of permanent landmarks at decisions points [29], underscore the potential importance of the stability or permanence of landmarks.

This not only makes intuitive sense – in order to build an environmental representation, stable features are clearly desirable – but landmark permanence has long been held to be a prerequisite for constructing effective cognitive maps [6]. Control of hippocampal place cells during cognitive map formation is known to be stronger when landmarks are stable [31]–[33]. Landmark permanence is not thought to be coded by the hippocampus directly, but rather hippocampal place cells may be guided by stability signals coming from elsewhere. The responsivity noted above of PHC and RSC during fMRI to attributes related to item permanence [29], [30] may make them candidate regions for coding landmark permanence. Further indirect evidence for this comes from Committeri et al. [34] (see also [35]), who observed PHC and RSC engagement when proximity judgements were made relative to enduring landmarks in a virtual environment. RSC is particularly interesting in this regard, as patients with RSC lesions, while still able to recognise landmarks, are unable to derive navigational information from them and so become disoriented [36], [37]. RSC contains head direction cells [38], [39], which may provide a mechanism for registering permanent landmarks, and anchoring neural responses to them for use in environmental representations. This might also be true of other regions known, at least in animals, to contain head direction cells such as anterodorsal thalamus and the postsubiculum [9], although evidence for the role of the latter two in human navigation is scarce.

In summary, while landmarks have been at the heart of empirical research and theoretical and computational models of navigation for decades, there is a surprising lack of direct information about the key attributes of landmarks and their neural substrates. We therefore set out to consider landmarks in a systematic manner, focussing specifically, and to our knowledge for the first time in an fMRI study, on landmark characteristics and the brain regions they engage. Based on the extant literature, the following features of landmarks were examined: their visual salience, their size, whether they were space-defining [30], their navigational utility, the permanence of landmarks, and their portability. There were three aspects to this study; first, in a set of behavioural experiments a large set of outdoor items were characterised for these attributes. This was followed by an fMRI study which utilised an optimised sub-set of these stimuli that covered a range of values for each landmark property, while also minimising any correlations between. Importantly, the participants in the fMRI study were naïve to our interest in landmarks and their properties, and during scanning merely viewed each image one at time and performed a vigilance task – pressing a button if a blue dot appeared on an item. The naivety of the fMRI participants, the incidental task, and the absence of manipulations related to navigation meant that we could conduct an unbiased and specific assessment of implicit and automatic neural responses to the landmark characteristics of interest. We hypothesised that PHC would be engaged by a range of the landmark features, given previous observations of its responsivity to landmarks at decision points, space-defining landmarks, large and more permanent landmarks [26], [29], [30], [34], [35]. By contrast we predicted that RSC (specifically BA 29/30, and possibly the anterodorsal thalamus/subicular region) might be particularly engaged by landmark permanence [29], [34], [35].

The final aspect of the study concerned individual differences. As alluded to, individuals can vary in their assessment of landmarks, and we wondered whether navigation ability could have an influence, and if so, whether this would be manifested in the brain regions engaged, thus providing further insights into the potential influence of landmarks in forming effective environmental representations.

## Results

### Characterising Landmark Properties

In order to investigate landmark features, we compiled a set of 683 images, each depicting a single, everyday, outdoor item devoid of additional context, shown on a white background. Forty-eight, healthy, right-handed participants (24 female, mean age 23 years, SD 2.90) took part in three experiments (16 participants - 8 females - in each experiment; see also Materials and Methods) in order to make ratings of these items along the following parameters:

1. *Navigational utility*: Would you use this if you were trying to find your way? (1) No (2) yes.
2. *Size*: What size do you expect the item in this picture would be in real life? (1) Very small (2) small (3) medium (4) large (5) very large.
3. *Visual salience*: To what extent do you think this would grab your attention? (1) Not at all … (5) very much.
4. *Space-defining or space ambiguous (SD/SA)*: Does this item rapidly evoke a sense of surrounding space? (1) Not space-evoking (2) space-evoking.
5. *Permanence*: How often would you expect the position of this item to change in everyday life? (1) Very often (2) often (3) occasionally (4) rarely (5) never. It was made clear to participants that this related to the overall landmark, and not to any (moving) parts of the landmark.
6. *Portability*: How easily do you think you could move this item? (1) Easily on my own (2) on my own with difficulty (3) with help from one other person (4) with help from multiple people (5) it’s not moveable.

Using these ratings, we selected an optimised set of 280 stimuli for use in the fMRI experiment (this number was the most that could be viewed within a reasonable time in the scanner). Selection was based upon consistency of responses for the features across at least 60% of participants, whilst ensuring a broad range of values for each attribute, given that we were interested in parametric responses. Most importantly, we also ensured that the final set of stimuli minimised the correlations between the item attributes. For example, items that were rated as permanent had a broad range of sizes, including many small and medium-sized permanent items as well as large permanent items.

A new set of 32, healthy, right-handed participants (16 female, mean age 23.5 years, SD 3.05), none of whom had taken part in any of the behavioural studies, participated in the fMRI study. They were naive to the purpose of the experiment, focussing instead on performing a vigilance task as they viewed each landmark in turn (see Materials and Methods). During a debriefing session after scanning, the participants were shown each landmark again and rated them along two permanence-related parameters:

1. *Permanence (post-scan)*: How often would you expect the position of this item to change in everyday life? (1) Very often (2) often (3) occasionally (4) rarely (5) never. As in the behavioural studies, it was made clear to participants that this related to the overall landmark, and not to any (moving) parts of the landmark.
2. *Distance moves*: How far would you expect this item to move in a normal day? (1) Over 10 miles (2) about 1 mile (3) about 100 metres (4) metres (5) centimetres.

This was only asked if the participant indicated in the previous question that the item could change position. This mix of imperial and metric ratings was found to be the most intuitive for participants.

Given that permanence ratings were made by the behavioural participants (rating number 5 above) and post-scan by the fMRI participants (rating number 7 above), we examined the correspondence between these two sets of ratings for the 280 scan stimuli. The ratings were highly correlated (r = 0.95, p<0.001); in addition, there was no significant difference in the mean scores (t_46_ = 0.810; p = 0.42). This confirmed that the ratings made by the behavioural and scan participants were comparable, and that the landmark characterisations made by the behavioural study participants were appropriate to use in the fMRI analyses.

**Figure 1:**
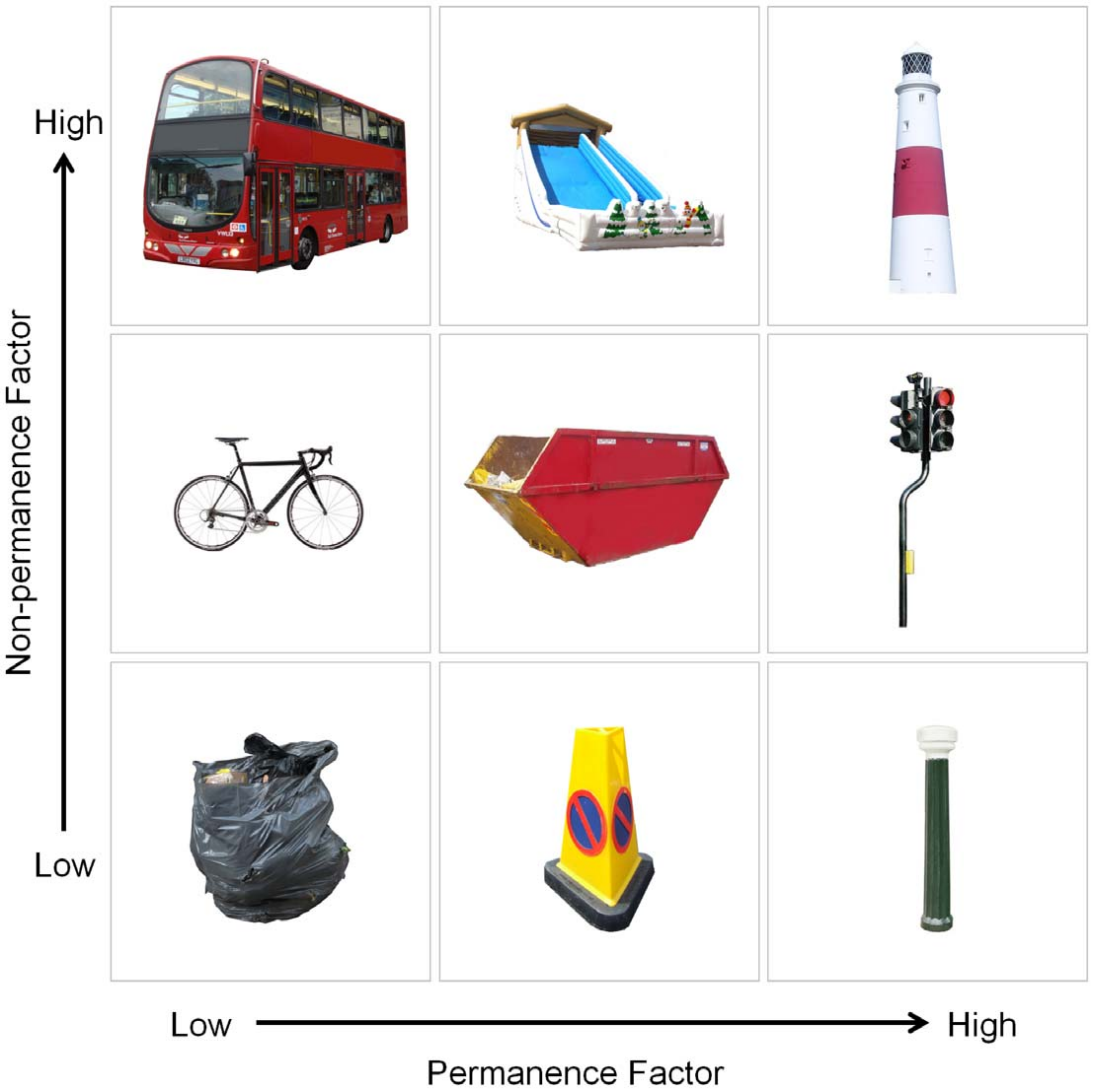
Examples of the stimuli. Example items are shown from the 280 stimuli used in the fMRI study. Level of permanence (from low to high) is shown from left to right. Shown vertically from bottom to top, variation (from low to high) in terms of the non-permanence factor. For further examples of the stimuli see Figure S2.

Because we had 8 separate measures of features for the 280 scan stimuli, we reasoned that some of these variables may potentially load onto common underlying components. We therefore submitted the scores to a principal components factor analysis using a varimax rotation and Kaiser normalization. Two factors accounted for 81.94% of variance in the data (see Table 1): navigational utility, size, visual salience, and SD/SA loaded strongly onto one (non-permanence) factor, while the permanence-related features - permanence, permanence (post-scan) and distance moves - loaded together on the second factor. Portability loaded similarly on both factors reflecting its relationship to size on the one hand and permanence on the other. Thus the factor analysis confirmed the presence of two key components in the landmark features that we assessed (see examples in Figure 1; see also Figure S2), and in particular highlighted permanence of landmarks as a distinct factor.

**Table 1:** Results of the factor analysis.

	Principal Components Analysis Loadings	
Landmark Feature	Factor 1	Factor 2
Navigational Utility	**0.787**	0.352
Size	**0.924**	0.043
Visual Salience	**0.722**	−0.144
SD/SA	**0.908**	0.105
Portability	**0.665**	**0.599**
Permanence	0.235	**0.908**
Permanence (post-scan)	0.124	**0.978**
Distance Moves	−0.174	**0.946**

### Neural Substrates of Landmark Properties

During scanning, the fMRI participants, who were naïve to our interest in landmarks, engaged in a vigilance task. They performed with a high level of accuracy (mean 93.7%; SD 8.75), showing they focussed on this dot-detection task and maintained attention during the experiment. The catch trials were removed from the fMRI analysis.

Our interest was in understanding the neural substrates of the landmark features, specifically, how the fMRI BOLD response reacted to changes in landmark attributes. In order to do this, we needed to take account of the fact that the landmark attributes shared some underlying components. For each stimulus we calculated orthogonal factor score coefficients for the factor analysis’ two principal components using the Anderson-Rubin method. Parametric regressors from these scores were then entered into a whole brain GLM fMRI analysis. This enabled us to examine activity that was linearly modulated by factor 1, and activity linearly modulated by factor 2.

**Figure 2:**
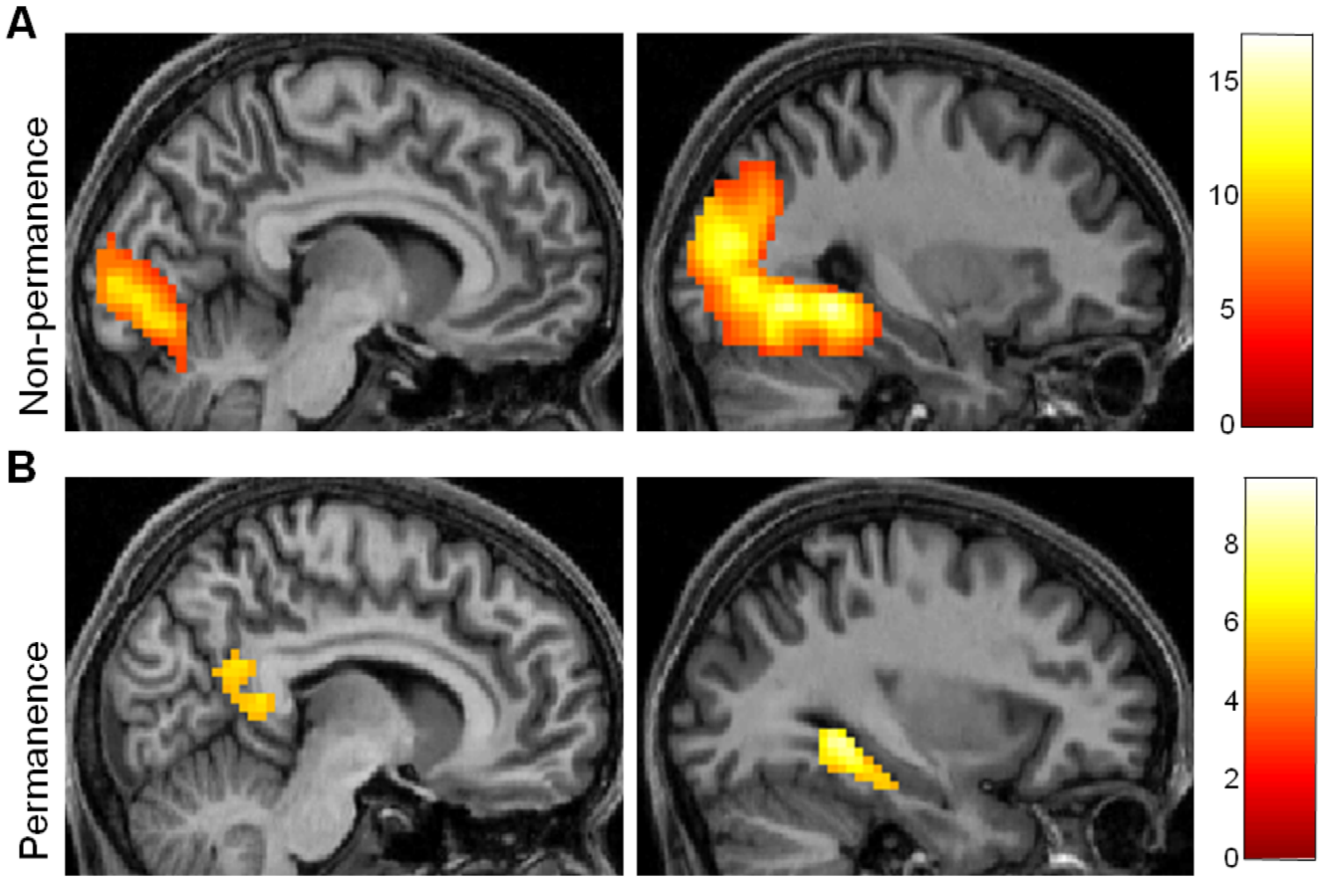
Brain regions engaged by the non-permanence and permanence components of the factor analysis. Activations are displayed on sagittal views of the structural MRI brain scan of one participant chosen at random. The colour bars indicate the Z-scores associated with each voxel. (A) The PHC and posterior visual areas were activated by increasing values of the non-permanence factor. (B) RSC, along with PHC, was activated by the permanence factor.

For increasing values of the first factor (which had high loadings for navigational utility, size, visual salience, and SD/SA) increased activity was present in right PHC (30, −46, −8; Z = >8) and left PHC (−27, −61, −8; Z = 7.74) extending posteriorly into right and left occipital cortex (15, −94, 4; Z = >8; −18, −85, −8; Z = >8). There were additional peaks in left cerebellum (−15, −49, −41; Z = 5.44) and left superior parietal cortex (−21, −64, 55; Z = 4.95) (see Figure 2A). Decreasing values of this factor were not associated with any changes in activity. Increasing scores for the second factor (which had high loadings for permanence, permanence (post-scan) and distance moves) were associated with increased activity in right PHC (30, −40, −5; Z = 6.44) and left PHC (−30, −43, −5; Z = 6.00), as well as in right RSC (9, −46, 10; Z = 4.79; 9, −52, 22, Z = 4.81) and left RSC (−9, 46, 7; Z = 4.82) (see Figure 2B). Decreasing values of this factor were associated with changes in activity in left and right occipital cortex (−18, −91, 1; Z = 5.93; 24, −88, −2; Z = 5.88). In summary, all of the landmark attributes (i.e. both factors) significantly engaged PHC. However, permanence related-features induced further strong activation in RSC (specifically BA 29/30).

**Figure 3:**
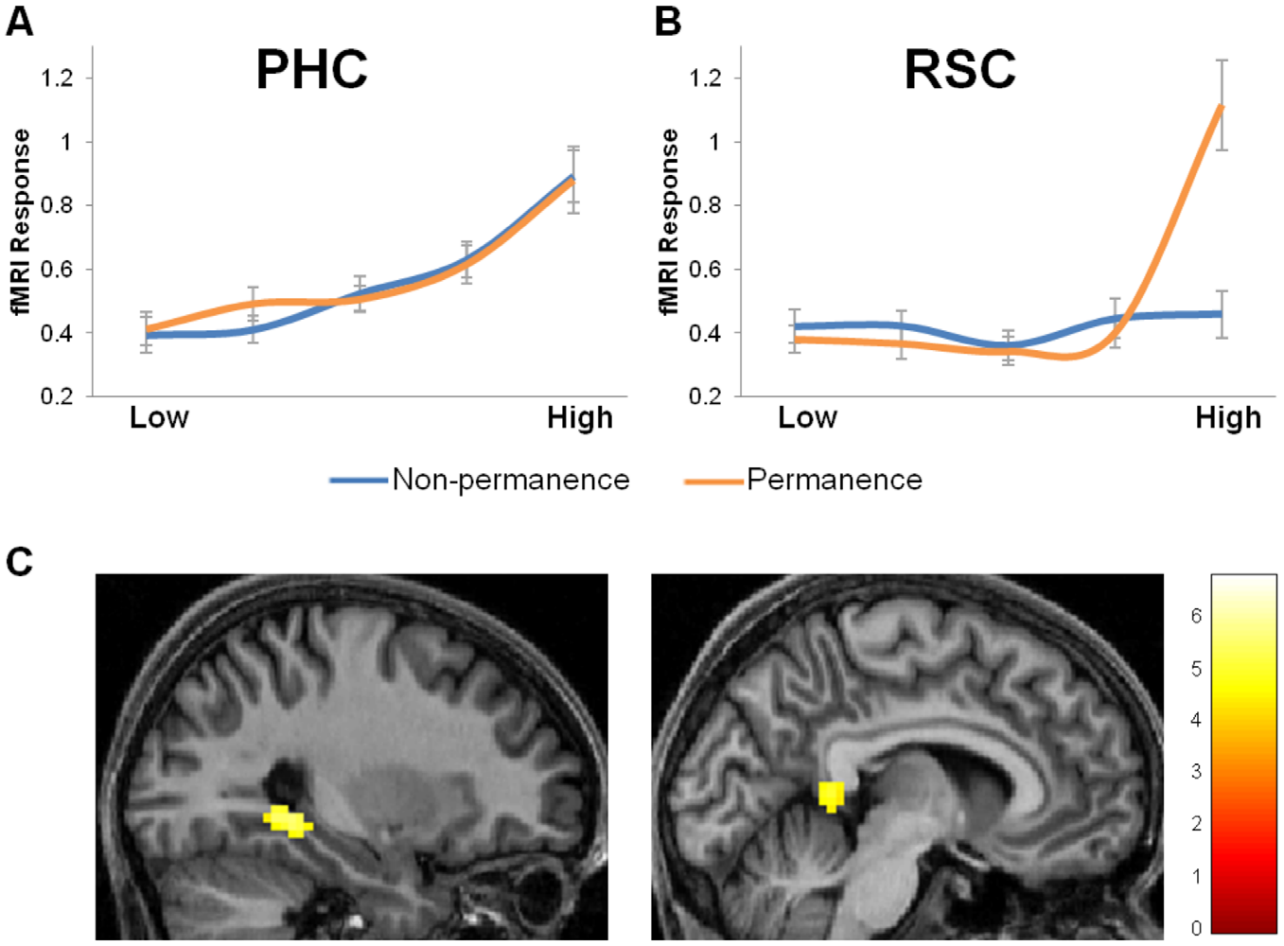
Response profiles of the PHC and RSC. The fMRI BOLD response to the non-permanence (blue) and permanence (orange) factors are shown for (A) the PHC and (B) the RSC. Mean scores are plotted +/−1 SEM. Landmarks were grouped into 5 bins according to the values of their factor score estimates, and these were approximately equivalent to the five rating values, e.g. for the permanence factor ‘low’ means landmarks that were not at all permanent, ranging to ‘high’ meaning permanent landmarks. Note that the response profiles of these two factors bore close relation to those of the individual features from which they were composed in the principal components analysis, and so provide a reliable summary of all the features. (C) Brain areas more active for landmarks rated as high compared to low in permanence. Activations are displayed on sagittal views of the structural MRI brain scan of one participant chosen at random. The colour bars indicate the Z-scores associated with each voxel.

We then conducted a second analysis focussed on anatomically-defined regions of interest (ROI) in PHC and RSC (see Materials and Methods). The fMRI BOLD response profiles for PHC and RSC for the two factors were extracted and plotted - see Figure 3. The PHC clearly responded to both factors, showing a linear increase in responsivity as the values for the factors increased (Figure 3A). This was not the case for RSC, where its activity did not change as a function of increasing value of the features loaded onto factor 1 (the non-permanence features). Furthermore, for the permanence-related landmark attributes loaded onto factor 2, its profile of response was not linear. Instead, what is quite apparent from Figure 3B is the large increase in RSC response specifically to the landmarks that were the most permanent. Indeed, comparing directly the landmarks rated as most permanent with those rated as least permanent in a whole brain fMRI analysis confirmed the engagement of the RSC (−6, −46, 4; Z = 4.22; and PHC: −30, −43, −5, Z = 5.28; 33, −37, −8; Z = 4.84) for the most permanent landmarks (Figure 3C).

In summary, the ROI analysis concurred with and extended the whole-brain results, showing that activity in PHC was influenced by parametric changes in a wide range of landmark properties, whereas RSC was sensitive specifically to the most permanent landmarks.

### The Effect of Navigational Ability

In this study we also explored whether navigation ability affected the characterisation of landmark properties, and how this might relate to fMRI responses.

At the end of the post-scan debriefing session, each of the 32 fMRI study participants completed the Santa Barbara Sense of Direction Scale (SBSOD; [40]). This is a self-report questionnaire that has been shown to correlate strongly with actual navigation ability, and is increasingly used as a reliable proxy for real-world wayfinding performance [27], [28], [40]. We defined two groups, good and poor navigators (n = 16 in each group) by taking a median split of SBSOD scores (mean for the good group 5.5, SD 0.56; the poor group 3.9, SD 0.62). The two groups were matched for age (mean age good navigators 23.5 years, SD 2.78; poor 23.6 years, SD 3.39; t_30_ = −0.057; p = 0.96), the proportion (good 92.6%, SD 9.83; poor 94.8%, SD 7.69; t_30_ = −0.713; p = 0.48) and speed (good 416 ms, SD 80.1; poor 456 ms, SD 81.5; t_30_ = −1.383; p = 0.18) of catch trial dot detection during scanning, their visual memory as measured by the delayed recall of the Rey-Osterrieth Complex Figure [41], [42] (good 20.9, SD 6.90; poor 19.4, SD 6.78; t_30_ = 0.63; p = 0.53), and their visual information processing ability and abstract reasoning skills as measured by the Matrix Reasoning sub-test of the Wechsler Abbreviated Scale of Intelligence [43] (mean scaled score good 12.2, SD 1.38; poor 11.6, SD 1.82; t_30_ = 1.09; p = 0.28). We also conducted a voxel-based morphometry (VBM; [44], [45]) analysis to investigate whether any structural brain differences were apparent between the groups. No differences in grey or white matter anywhere in the brain, including in PHC and RSC, were detected. While the groups were matched for gender (8 female in each group), we also analysed all of the behavioural, VBM, and fMRI data to compare males and females directly, and did not find any significant differences between the sexes. Thus, the only evident difference between the good and poor navigators was in their declared navigation ability.

**Figure 4:**
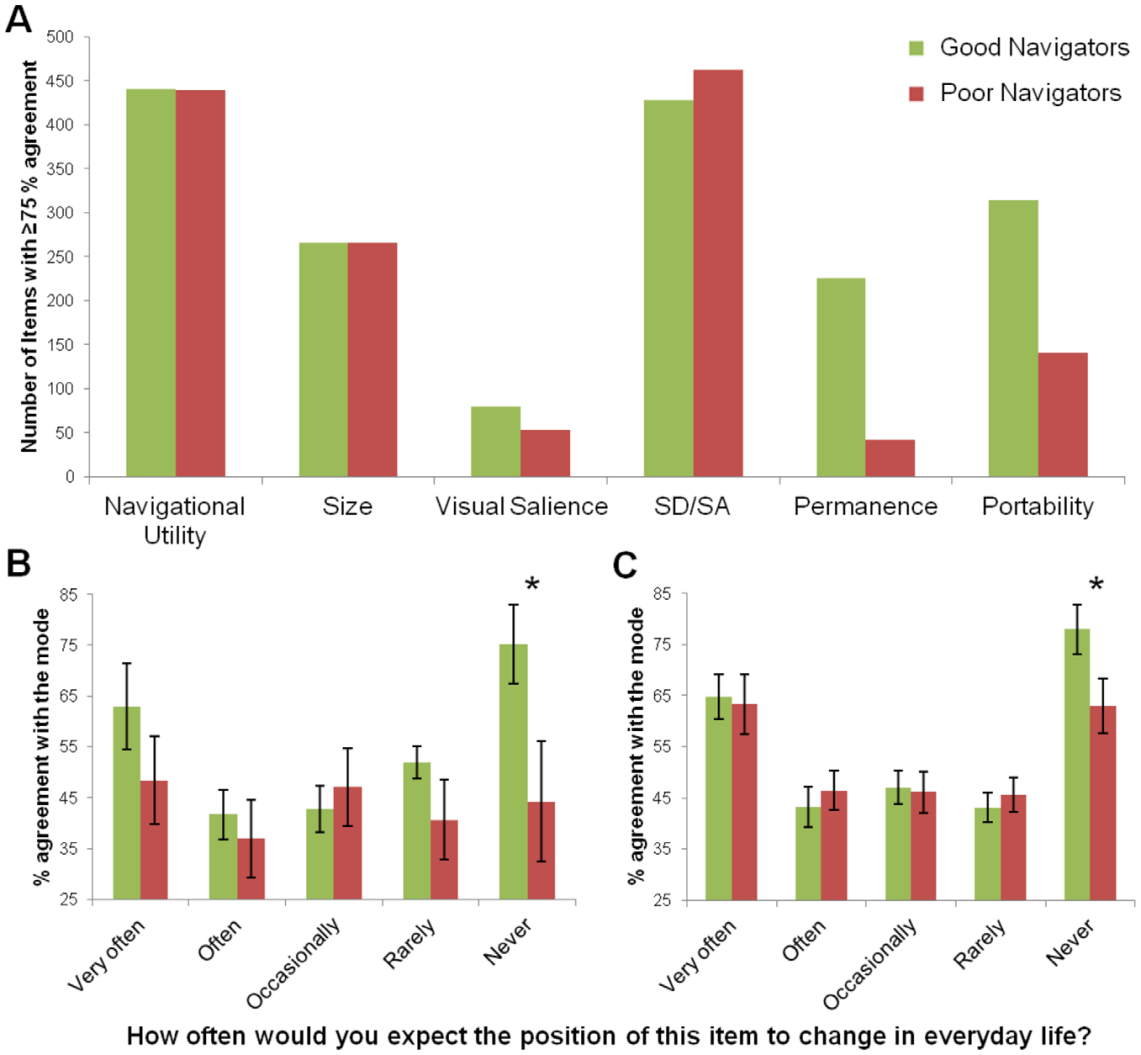
Landmark feature ratings segregated according to navigation ability. Good navigators are shown in green and poor navigators in red. (A) The number of landmarks where at least 75% of participants within each group gave the same rating. It is clear that the only difference between good and poor navigators was for permanence and portability. (B) Focussing on the permanence ratings, we examined how often each participant gave a rating which was different to the most common rating for each item (i.e. the mode). Good and poor navigators did not differ in rating items which were most commonly scored 1 to 4 for permanence, however, there was a significant difference between the groups for rating number 5, landmarks that were the most permanent and never moved. (C) This difference for the most permanent landmarks was replicated in the independent group of fMRI participants. *P<0.05; graphs show the means +/−1 SEM.

In order to examine whether navigation ability had an effect on the processing of landmark attributes, we re-examined the ratings participants gave for the landmarks, now taking their navigation ability into account. We first looked at how much overall agreement there was among good and poor navigators in the first set of behavioural studies (these participants also completed the SBSOD questionnaire) in scoring the different features of the original 683 landmarks. Examining the number of landmarks where at least 75% of participants within each group gave the same rating, there were no clear differences between good and poor navigators in the number of high consensus items for navigational utility, size, visual salience, or SD/SA. However, for ratings of permanence-related features of landmarks, there was a large discrepancy between the amounts of agreement within the groups (see Figure 4A), with much greater consensus about the permanence and portability of landmarks among the good navigators and much less among the poor navigators.

**Figure 5:**
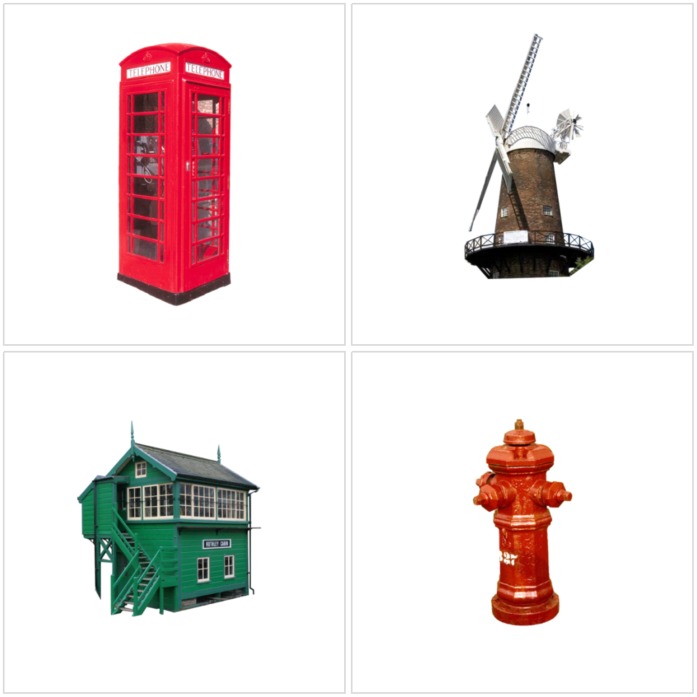
Examples of landmarks where good but not poor navigators had at least 75% agreement about their ‘never moves’ permanence rating.

We then examined the permanence ratings in more detail; as a reminder, the permanence question that participants answered was: How often would you expect the position of this item to change in everyday life? (1) Very often (2) often (3) occasionally (4) rarely (5) never. We looked at how often each participant gave a rating which was different to the most common rating for each item (i.e. the mode). We found that good and poor navigators did not differ in rating items which were most commonly scored 1 to 4 for permanence, however, there was a significant difference between the groups for rating number 5, landmarks that were the most permanent and never moved (t_14_ = 2.183; p = 0.047; see Figure 4B). To assess the robustness of this finding, we also examined the post-scan permanence ratings for the 280 scan stimuli provided by the independent group of 32 participants who took part in the fMRI component of the study. Here too, the only difference between good and poor navigators was for the most permanent landmarks (t_30_ = 2.082; p = 0.046; see Figure 4C). Interestingly, there were no differences between the groups for any of the ‘distance moves’ ratings, including for landmarks that were rated to move by only centimetres (t_30_ = −0.412; p = 0.68), further underlining the specificity of the good-poor navigator difference only for items which truly never move. Examples of landmarks where good, but not poor, navigators had at least 75% agreement about their ‘never moves’ permanence rating, are shown in Figure 5.

**Figure 6:**
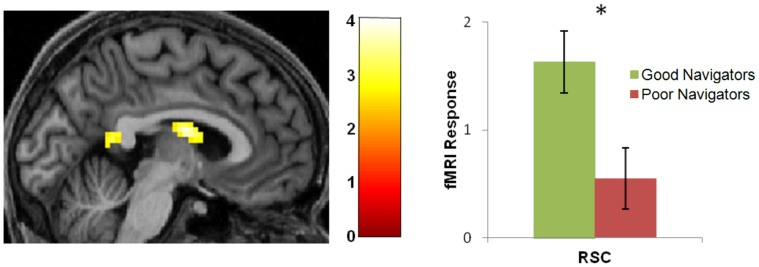
Brain regions more active in good than poor navigators when viewing the most permanent landmarks. (A) Good navigators had greater activity in RSC and anterodorsal thalamus than poor navigators when viewing the most permanent items but not the less permanent ones. Activations are displayed on sagittal views of the structural MRI brain scan of one participant chosen at random. The colour bars indicate the Z-scores associated with each voxel. (B) The mean (+/−1 SEM) response in active RSC voxels to the most permanent items was significantly greater in good (green) than in poor (red) navigators. *P<0.05.

As the behavioural difference between good and poor navigators was driven by the most permanent landmarks, in a whole brain fMRI analysis we directly contrasted good and poor navigators focussing specifically on the landmarks that never moved. We observed significantly greater activity in RSC (−3, −49, 13, Z = 2.83) when good navigators viewed these most permanent landmarks than when poor navigators viewed them (Figure 6A). There was also significantly greater activity in good navigators in the anterodorsal thalamus (0, −4, 13; Z = 3.87). Figure 6B shows the mean response of active voxels in RSC for good and poor navigators for the most permanent items, with a significantly higher response in the good navigators. There were no differences in any other brain regions, including the PHC, and no brain areas were more active for poor navigators. We also compared the good and poor navigators for the other permanence ratings and found no differences between the groups for the ratings 1–4 either separately or combined.

In summary, good and poor navigators, who were matched on a range of demographic, cognitive and structural brain measures, differed not only in their navigational ability, but in two other ways. Poor navigators had: (1) considerably less agreement when identifying the most permanent landmarks (but not any other features), a finding replicated across two independent samples of participants; and (2) significantly reduced activity in RSC and anterodorsal thalamus specifically in response to landmarks that were most permanent.

## Discussion

There were three key findings from this study. First, focusing on a range of landmark attributes, we ascertained that these features were underpinned by two components, which included the permanence of landmarks. Second, while we observed parametric responses in parahippocampal cortex to increasing values of both components, activity in retrosplenial cortex (BA 29/30) responded specifically to the most permanent landmarks. This is interesting because the role of the RSC is somewhat mysterious. Known to be involved in supporting scene processing [18], navigation [36], [37] and autobiographical memory [17], [46], [47], there is little agreement about what its primary function might be. By revealing here its responsivity to landmark permanence, this could represent an intriguing new way of conceptualising its contribution. The third finding from our study provides further support for the relationship between the RSC and landmark permanence. We found that in two independent cohorts, poor navigators, relative to good navigators, made less reliable decisions about landmark permanence, specifically for the most stable landmarks. Moreover, this was accompanied by reduced RSC activity when poor navigators viewed the permanent landmarks. This offers a novel insight into a possible reason for poor navigation ability in some individuals. If a person cannot register effectively the most stable features in an environment, then the resultant internal representation of that environment may be less reliable and more likely to produce disorientation.

Landmark properties have received surprisingly little direct attention in navigation neuroscience, despite being potentially informative about how environmental representations are formed and supported. Nevertheless, the permanence of landmarks has been noted to influence the control of hippocampal place fields in rats and the stability of resultant cognitive maps [6], [10], [31]–[33]. The question of where landmark permanence is itself coded has not been addressed directly. Our findings show that the human RSC responds specifically to the most stable landmarks. Given its strong connectivity with the hippocampal region [37], [48]–[51], information about the permanence of landmarks that is coded in RSC may be shared with the medial temporal lobes, contributing to the formation of environmental representations. This view is compatible with the observation that temporary inactivation of the rat RSC transiently alters the spatial tuning of hippocampal place cells [52]. Moreover, several animal navigation studies have linked the RSC to processing behaviourally-significant and predictive environmental cues [53]–[58]. Thus, the presence of stable landmarks/cues in any spatial experiment may engage or require the RSC.

Lesions to the RSC in rodents impair spatial navigation [37]. While the nature of the tasks varies, it is interesting to note that many of them involved fixed or more stable (distal) cues although, to our knowledge, the effect of RSC lesions on landmark/cue permanence per se has not been explicitly examined. In humans, too, landmark permanence has not been tested in the context of RSC damage. The consistent finding from such patients is, as with the animal data, one of disorientation [36], [37]. Based on our findings we suggest that this disorientation could result from a failure to identify reliable stable landmarks from which to derive navigational information. If patients with RSC lesions are unable to identify the most permanent, stable cues in an environment, then their resulting representations will be disordered, adversely affecting navigation in both familiar and new environments. This may in part also explain the spatial disorientation experienced by those with Alzheimer’s dementia, given that RSC hypometabolism has been observed in the earliest stages of the disease [37], [59].

To some extent this may be also the case in our poor navigators. They were matched to the good navigators on every measure – demographic, cognitive, and in terms of brain structure. There was also no significant difference between the two groups when making any of the ratings, including ratings of distance moves (even when items were rated to move by only centimetres). The two groups differed solely in the decisions they made about the most permanent landmarks, where the poor navigators in particular could not agree. Examples of these are provided in Figure 5, and are quite surprising. For instance, how can a building be regarded as anything but permanent? Yet this result was replicated in two independent samples of participants, underlining the robustness of the finding. Alongside this misidentification of the most permanent landmarks, the poor navigators also had a reduction in RSC fMRI BOLD response specifically to the most permanent landmarks. This difference was only apparent for RSC and not for PHC. We believe this is further compelling evidence that the RSC codes for the most permanent landmarks, and this might be its fundamental contribution to spatial navigation. It is notable that good and poor navigators did not differ when rating the navigational utility of landmarks. It seems, therefore, that while participants, even poor navigators, had high agreement about what was likely to be navigationally useful, in practice, effective navigation may be more reliant on landmark features such as permanence.

The retrosplenial region has been reported to be more engaged by familiar compared with unfamiliar landmarks, or with increasing familiarity of landmarks and spatial layout during learning (e.g. [60]–[62]), which seems difficult to reconcile with our permanence finding. However, in those studies the regions activated do not in fact correspond to RSC (BA 29/30) but are located more posteriorly and superiorly in posterior cingulate cortex, which is known to be activated during recollection [63]). It has also been suggested that the role of the RSC is one of translation between egocentric and allocentric frameworks (reviewed in [37]), although direct evidence for this is lacking. That RSC might in fact be primarily concerned with coding the most permanent landmarks is not necessarily at odds with a translation account. The identification of permanent landmarks could be viewed as an intermediate between egocentric experience of the environment and then the use of landmark permanence information in allocentric spatial representations. In other frameworks, emphasis has actually shifted away from landmarks as the basis for environmental representations, with boundaries and other terrain features instead being regarded as key [8], [64]–[67]. In the real world, however, boundaries are often comprised of landmarks, e.g. large buildings, whereas this is not typically the case in rat enclosures. Indeed, the pre-eminence of boundaries in cognitive maps has been questioned, with Lew [10] arguing that the apparent importance of boundaries may in fact relate to underlying properties such as their general stability during navigation, which resonates with our findings.

The mechanism for registering permanent landmarks may involve head direction cells, which are present in the RSC [38], [39], anchoring themselves to the most permanent landmarks. It is notable that, along with the RSC, the anterodorsal thalamus was also more active in the good compared to the poor navigators for the most permanent landmarks. The anterodorsal thalamus is heavily connected with the RSC [37] and head direction cells are also present there [68]. Damage to this region is known to cause spatial learning and memory impairments [69], and along with the RSC and hippocampus, the thalamus is thought to form a key circuit for spatial memory and recollection [37], [69]. Interestingly, we did not observe engagement of subicular regions or the hippocampus. Our task did not involve active navigation, instead the participants during scanning merely performed a vigilance task while viewing single, isolated landmarks. Overall, this suggests that RSC and anterodorsal thalamus may be automatically and rapidly deployed at the earliest stages of processing items that have relevance for navigation. The output of this process may then be made available upstream to other medial temporal regions in the navigation system.

The other clear component to emerge in our factor analysis comprised features such as landmark size, whether they were space-defining, their navigational utility, and their visual salience. Unlike the permanence factor, this component seems to reflect general visual properties of the items. Many fMRI studies report co-activation of PHC and RSC, and it has been a challenge to differentiate their individual contributions. Here, we observed the highly specific engagement of RSC for only the most permanent landmarks. By contrast, activity in the PHC parametrically increased for both non-permanent and permanent factors. This accords with the previous findings where PHC responded to space-defining landmarks which comprised large and permanent items [30], and objects at navigationally-useful decision points [26]–[29]. Interestingly, PHC activity did not differ between good and poor navigators, even for the most permanent landmarks, suggesting that PHC, unlike RSC, is not specifically concerned with the most stable landmarks. Instead, PHC appears to be involved in processing a broader range of generic object characteristics (e.g. object size and space-defining quality [30]) indicative perhaps of a more general role in the construction and processing of spatial representations.

In conclusion, our results provide further evidence that despite being labelled as ‘scene-selective’ cortex [70]–[75], PHC and RSC do not in fact require scenes in order to be engaged, instead activating strongly in response to features of single isolated landmarks (see also [30]). By revealing the specific engagement of RSC in response to the most permanent landmarks, this may help to explain the ubiquity of RSC activations in fMRI studies not only involving scenes and navigation, but also autobiographical memory [46], [47] and thinking about the future [76], [77]. Scenes, environments to be navigated, and real and imagined experiences all have a background context. Activation of the RSC in such instances may simply (but crucially) reflect the processing of permanent features in those scenes or events, thus helping to (re)construct a stable backdrop. Overall, our results highlight the need for further studies in humans and non-humans that focus on landmarks. Moreover, future studies should seek to establish precisely how the RSC comes to code for the most permanent landmarks, and the full extent of its influence on the ability to navigate successfully.

## Materials and Methods

### Participants

The 48 participants in the behavioural studies and the 32 participants in the fMRI study (details are provided in the main text) were all healthy, right-handed, highly proficient in English, and had normal or corrected to normal vision.

### Ethics

Ethics approval for this study was obtained from the University College London research ethics committee. All subjects gave informed written consent to participation in accordance with the approval of this ethics committee.

### Stimuli

The images used in the behavioural and fMRI experiments were all the same resolution and occupied a similar portion of the screen. We also examined the spatial frequency of the stimuli. To verify that this low level visual property was not driving the effects we observed, we performed an additional analysis where we included this in the factor analysis. Spatial frequency did not load strongly on either the non-permanence or permanence factors, confirming that it did not influence our findings.

### Procedure: Behavioural Studies

In the three experiments (each lasting approximately two hours per participant), two different features of each item were rated. In the first experiment participants rated the permanence and then the portability of each item. In the second experiment participants rated each item’s navigational utility, and then its visual salience. In the third experiment participants evaluated the SD/SA nature of the items, and then gave ratings of their size. At the end of each experiment, participants completed the Santa Barbara Sense of Direction (SBSOD) questionnaire.

### Procedure: FMRI Study

Before entering the scanner, participants were informed they were being tested for vigilance and attention. They would be shown images of everyday outdoor items. They were instructed that a blue dot could appear anywhere on an image at any time and that they should respond with a button press as soon as they saw one. They were told to look closely at each image to ensure that they would not miss any of these dots. It was also stressed that participants should focus on the items and should not think about other objects, contexts or personal memories. Participants then practised the task using stimuli not included in the experiment proper. During scanning, the 322 images (280 plus 42 catch trial stimuli) were shown centrally on the screen, one at a time for 3 seconds each, with a randomly jittered interval of between 2 and 5 seconds separating trials, during which a black central fixation cross was displayed on a white background. The catch trials, during which a small blue dot appeared somewhere on a landmark image for 1 second, occurred randomly during the scanning sessions (of which there were three). No stimuli were repeated. The order of trials was pseudo-randomised with the proviso that landmarks with different values for the numerous features were distributed across the scanning sessions and that there were no systematic patterns in the presentation order. Immediately after scanning in a debriefing session, participants saw each stimulus again and made ratings of permanence and the distance they might move, as well as completing some neuropsychological tests (see Results section for details), and the SBSOD questionnaire.

### Scanning Parameters and Preprocessing

T2*-weighted echo planar images (EPI) with blood oxygen level-dependent (BOLD) contrast were acquired on a 3T whole body MRI scanner (Magnetom TIM Trio, Siemens Healthcare, Erlangen, Germany) operated with the standard RF transmit body coil and 12-channel head receive coil. Scanning parameters were selected to achieve whole brain coverage and optimised for the hippocampus and surrounding tissue: 48 oblique axial slices angled at −45° from the axial to coronal plane (as defined in [78]), 2.5 mm thickness (with inter-slice distance factor 20%), repetition time TR = 3.36s (slice TR = 70 ms), excitation flip angle  = 90°, echo time TE = 30 ms, in-plane resolution 3 mm×3 mm, field of view FoV  = 192 mm×192 mm, 64×64 matrix, phase encoding (PE) in the anterior-posterior direction, 13% oversampling in the PE direction, echo spacing 500 µs. For reduction of signal loss in the hippocampal region, slices were angulated and a z-shim gradient moment of +0.6 mT/m*ms was applied [78]. The first 6 ‘dummy’ volumes from each session were discarded to allow for T1 equilibration effects. Field maps were acquired with a standard manufacturer’s double echo gradient echo field map sequence (short TE = 10 ms, long TE = 12.46 ms; 64 axial slices with 2 mm thickness and 1 mm gap yielding whole brain coverage; in-plane resolution 3 mm×3 mm). A 3D MDEFT T1-weighted structural scan [79] was acquired for each participant with 1 mm isotropic resolution. FMRI data were analysed using SPM8 (www.fil.ion.ucl.ac.uk/spm). Images were realigned and unwarped (using the field maps), normalised to a standard EPI template in MNI space with a resampled voxel size of 3×3×3 mm and smoothed using an 8 mm FWHM Gaussian kernel.

### Scanning Data Analysis

#### Whole brain fMRI

Each trial was modelled from the time of onset of the stimulus for 1.5 seconds. This time period was selected as we were most interested in rapid and automatic responses to the stimuli. The mean rating for each of the 8 features of all 280 scanning stimuli was used in the principal components analysis. A separate regressor was created for catch trials, and was treated as a covariate of no interest, as were individual movement parameters. Regressors were convolved with the haemodynamic response function. Subject-specific parameter estimates pertaining to each regressor of interest (betas) were calculated for each voxel. Second level random effects analyses were then run using one-sample t-tests on these parameter estimates (collapsed across sessions). The categorical contrast of most versus least permanent landmarks compared items that had been given a post-scan permanence rating of 5 with those rated 1 or 2. We report all of the fMRI activations that survived a whole brain uncorrected threshold of p<0.001 (minimum cluster size of 5 voxels) for PHC and RSC, given our apriori interest in these brain areas, and p<0.05 (FWE corrected) for the rest of the brain. For good and poor navigators, the second-level analysis comprised a two sample t-test with FWE correction (p<0.05) using an RSC anatomical ROI (see below), p<0.001 whole brain uncorrected threshold for other navigation-relevant brain areas (see Introduction), and p<0.05 (FWE corrected) for the rest of the brain.

#### ROI

Anatomical masks for the PHC and RSC (defined as BA 29/30) were delineated by an experienced researcher not involved in the project on an averaged structural MRI brain scan from different set of n = 30 participants, and guided by Duvernoy [80] and Vann et al. [37]. Responses for the two factors were plotted by grouping stimuli into 5 bins (approximately equivalent to the five rating values) according to the values of their factor score estimates. Subject-specific parameter estimates pertaining to regressors for each of these bins were calculated for each voxel. For each bin, contrast values in active voxels (i.e. those with a value greater than 0) were averaged in the PHC and RSC regions, collapsing across left and right (given that responses in the two hemispheres were very similar) using the MarsBaR toolbox and then plotted.

#### VBM

Structural MRI scans were analysed using VBM implemented in SPM8, employing a smoothing kernel of 8 mm full width at half maximum. Good and poor navigator groups were directly compared using a two-sample t-test, and a whole brain uncorrected threshold of p<0.001 for the PHC and RSC, and p<0.05 (FWE corrected) for the rest of the brain.

## Supporting Information

Figure S1 **Preliminary evidence for RSC engagement by item permanence.** Unpublished data from [30] showed preliminary evidence for an association between RSC activity and item permanence.
(DOCX)

Figure S2 **Further examples of the stimuli.**
(DOCX)
