# Supplementary material for: Retrosplenial Cortex Codes for Permanent Landmarks
Source: PLoS One. 2012 Aug 17;7(8):e43620. doi: 10.1371/journal.pone.0043620 (PMC3422332; doi:10.1371/journal.pone.0043620)
Supplement: Figure S1 — Preliminary evidence for RSC engagement by item permanence. Unpublished data from [30] showed preliminary evidence for an association between RSC activity and item permanence. (DOCX) [file pone.0043620.s001.docx]

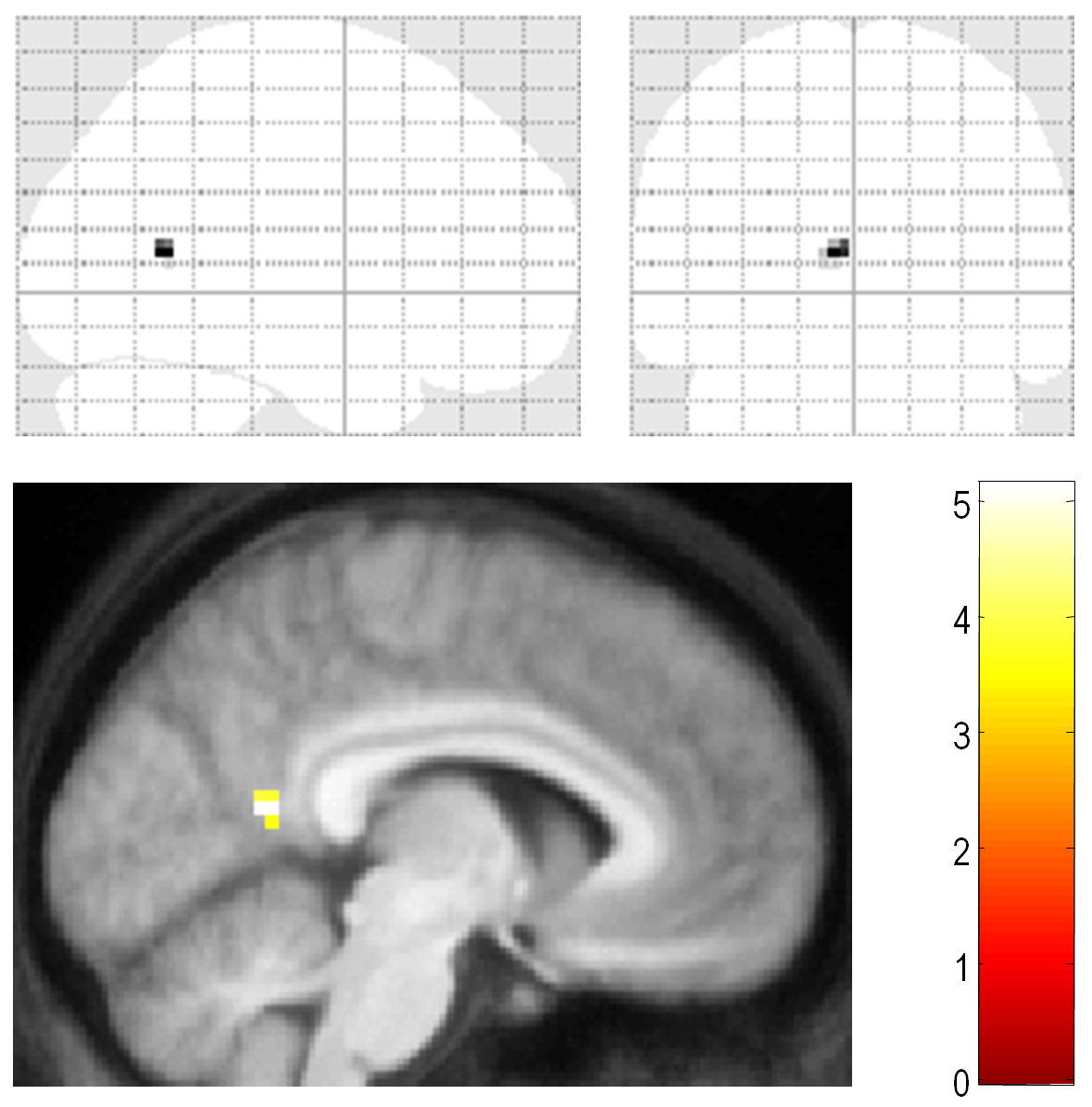


Figure S1. Preliminary evidence for RSC engagement by item permanence (unpublished data from [1]). Mullally and Maguire [1] previously reported a robust activation in bilateral parahippocampal cortex when participants visualised or viewed single space-defining objects (i.e. objects that consistently evoked a strong sense of the surrounding space) compared with space-ambiguous objects (i.e. objects that did not). Moreover, this response appeared to be driven by a combination of the objects’ size and their permanence within the environment. Interestingly, when the independent component of object permanence was explored, a highly selective response in left retrosplenial cortex was apparent (-6, -57, 12; Z = 4.06; p < 0.001, whole brain, uncorrected). This result suggests that the retrosplenial cortex may be particularly engaged by items that are more permanent and therefore stable within an environment. These data arose from a task where participants actively imagined isolated indoor objects which are rarely entirely stable within their environment (in the same way that buildings are stable in the outdoors). Its relevance to outdoor landmarks is, therefore, unexplored. Moreover, the discrete levels of object permanence were not fully balanced in this design (as this was not the primary goal of the experiment). In spite of these issues, this finding suggests a possible link between item permanence and the retrosplenial cortex, helping to motivate the current study.

[1] Mullally SL, Maguire EA (2011) A new role for the parahippocampal cortex in representing space. J Neurosci 31: 7441-7449.
