# Supplementary material for: Retrosplenial Cortex Codes for Permanent Landmarks
Source: PLoS One. 2012 Aug 17;7(8):e43620. doi: 10.1371/journal.pone.0043620 (PMC3422332; doi:10.1371/journal.pone.0043620)
Supplement: Figure S2 — Further examples of the stimuli. (DOCX) [file pone.0043620.s002.docx]

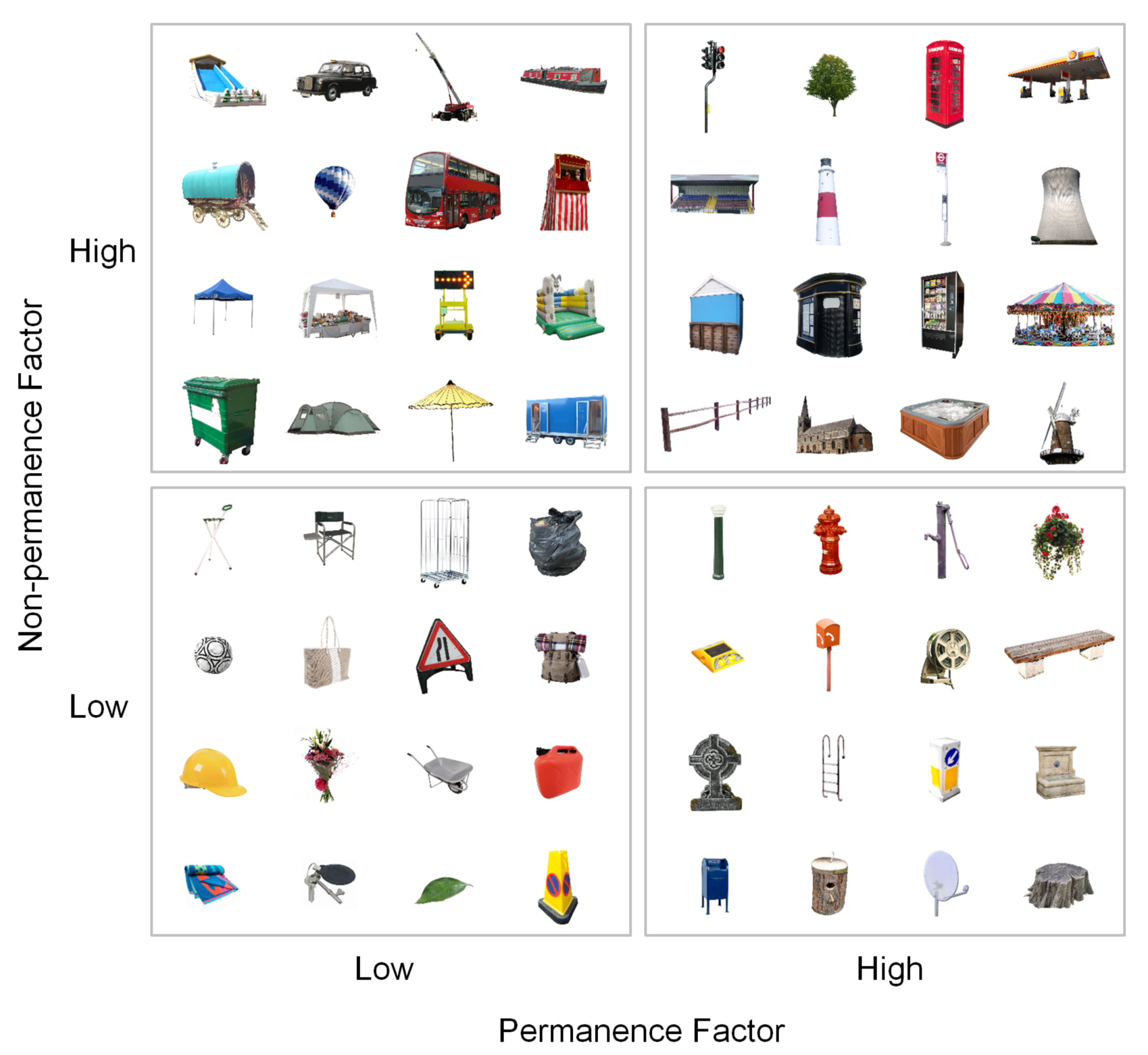


**Figure S2. Further examples of the stimuli.** Two factors emerged from the formal factor analysis that explained more than 80% of the variance in the stimulus set. Navigational utility, size, visual salience, and SD/SA loaded strongly onto one (‘non-permanence’) factor, while the permanence-related features - permanence, permanence (post-scan) and distance moves - loaded together onto a second (‘permanence’) factor. Examples of stimuli that scored low in permanence, i.e. moved their position a lot, are shown to the left of the figure while those that scored high in permanence, i.e. never changed their position, are shown to the right. Items that were scored highly on the non-permanence factor are shown in the upper part of the figure, and those that had low scores on this factor are show in the lower part of the figure.
